# Photoelectrochemical Imaging of Charge Separation between MoS_2_ Triangles and Insulating SiO_2_ Support

**DOI:** 10.1021/jacs.5c02136

**Published:** 2025-04-23

**Authors:** Ziyuan Wang, Qing Huang, Chenwei Ni, Tianyu Bo, Fengtao Fan, Michael V. Mirkin

**Affiliations:** † Department of Chemistry and Biochemistry, Queens College-CUNY, Flushing, New York 11367, United States; ‡ State Key Laboratory of Catalysis, Dalian National Laboratory for Clean Energy, iChEM, Dalian Institute of Chemical Physics, Chinese Academy of Sciences, Dalian 116023, China; § University of Chinese Academy of Sciences, Beijing 100049, China; ∥ The Graduate Center of CUNY, New York, New York 10016, United States

## Abstract

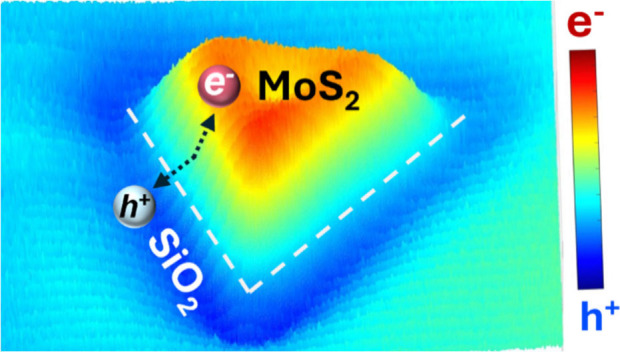

The role of the insulating support in photocatalysis is poorly understood. Using high-resolution photo-scanning electrochemical microscopy (photo-SECM), we observed significant spatial charge separation in few-layer-thick molybdenum disulfide (MoS_2_) triangles attached to a SiO_2_ substrate. Spatially resolved surface photovoltage (SPV) measurements revealed that photogenerated holes migrate from MoS_2_ to the SiO_2_ surface and travel laterally over distances exceeding 2 μm, driven by the built-in electric field of ∼1.7 kV/cm. In thicker and less uniform flakes, the charge separation is dominated by internal driving forces within MoS_2_, without significant contribution from SiO_2_. These findings underscore the importance of insulator–semiconductor interactions for effective charge separation, suggesting a new strategy for optimizing photocatalytic systems.

Photocatalysis is a crucial technology for converting solar energy and generating clean chemical fuels.^1,2^ In photocatalytic systems, particulate or thin-layer semiconductors are often attached to an insulating support, such as glass or SiO_2_.^3,4^ Domen and colleagues recently demonstrated that depositing a SiO_2_ layer on a photocatalyst surface can enhance the water-splitting efficiency by suppressing the backward oxygen reduction reaction.^5^ However, to the best of our knowledge, no systematic study of insulator–photocatalyst interactions has been reported to date. The studies of field-effect transistors revealed that the surface conductivity of insulators can be significantly enhanced by adsorption of polar molecules.^6−8^ This phenomenon was attributed to electron hopping between adsorbed sites across bare surface regions.^6,7^ Similarly, a hysteresis in conductance of graphene on a SiO_2_ substrate was caused by water adsorption and charge injection into trap states of the SiO_2_ substrate.^9,10^ Charge transfer was observed between two reduced graphene oxide sheets through insulating substrates such as mica, SiO_2_, and glass.^11^ The accumulation of electrons on insulating surfaces has been observed and used to conduct electrochemical reduction reactions, including electrodeposition of metals.^12^ These findings highlight the complex nature of insulator surfaces, where surface conductivity can be greatly influenced by external factors and enable two-dimensional charge transport.

Photo-scanning electrochemical microscopy (photo-SECM) is a powerful tool for studies of photocatalysis, enabling the detailed spatial mapping of photocatalytic activity and offering valuable insights into surface reactions under operando conditions.^13−20^ Here, we used photo-SECM to image photogenerated charge separation between few-layer-thick MoS_2_ triangles and the SiO_2_ support. Our results revealed that photogenerated holes migrate to the SiO_2_ substrate, whereas photogenerated electrons remain on the MoS_2_ surface. The spatial separation of photogenerated charges is driven by a strong built-in electric field at the SiO_2_–MoS_2_ interface.

Photo-SECM experiments were conducted in the feedback (Figure 1A-i) and redox competition (Figure 1A-ii) modes using a home-built setup for through-tip illumination of the sample, as described previously.^21^ The solution contained ferrocene methanol (Fc) redox mediator, and the tip potential (*E*_T_ = 0.4 V vs Ag/AgCl) was sufficiently positive for Fc oxidation. The substrate comprised few-layer MoS_2_ triangles CVD-grown on the SiO_2_ support. When the separation distance between the tip and substrate (*d*) was small enough (i.e., comparable to the tip radius, *a*), Fc^+^ produced at the Pt tip surface was reduced at the illuminated MoS_2_ surface, and the tip current (*i*_T_) increased with decreasing *d* (positive feedback). When the tip approached the insulating SiO_2_ substrate (or the MoS_2_ surface in the dark), the diffusion of Fc to the electrode surface was physically hindered, leading to a decrease in current with decreasing *d* (negative feedback). A negative feedback approach curve (*i*_T_ vs *d*) was fitted to the theory (Figure S1) to determine *a* = 150 nm.

**Figure 1:**
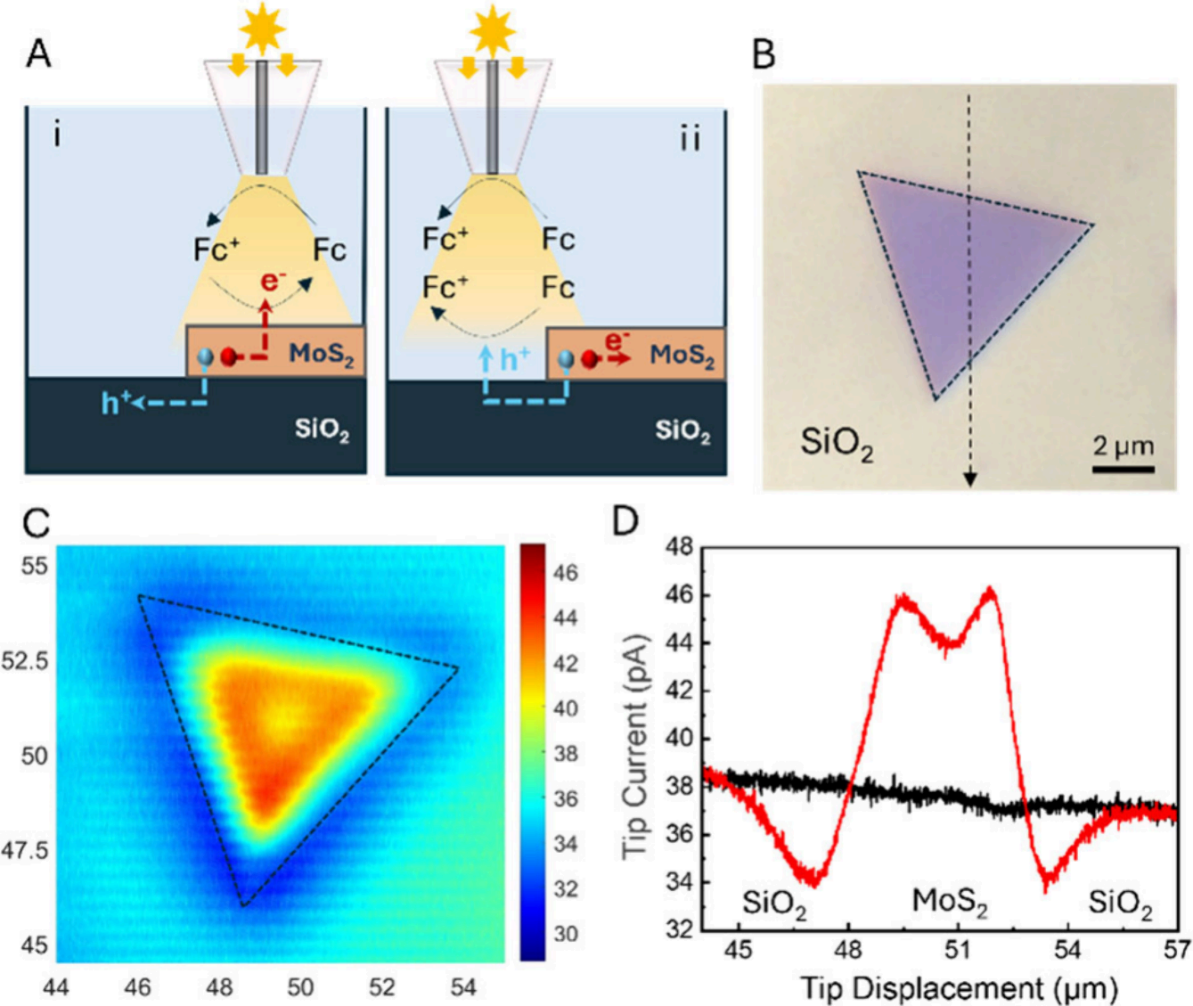
Photo-SECM of a MoS_2_ triangle. (A) Schematic representation of the positive SECM feedback over the illuminated MoS_2_ surface (i) and redox competition over the SiO_2_ surface adjacent to the triangle (ii). (B) Optical micrograph of a 5L MoS_2_ triangle. (C) Constant-height photo-SECM image of the same triangle. (D) Lateral scans along the specified trajectory (black line in panel B) under illumination (red curve) and in the dark (black). Solution contained 1 mM Fc in 0.1 M phosphate buffer (pH 7). *E*_T_ = 0.4 V vs Ag/AgCl. *a* = 150 nm.

Figure 1B,C shows an optical micrograph (B) and a photo-SECM image (C) of the same ∼4 nm thick five-layer (5L) MoS_2_ triangle (Figure S2A). The SECM feedback is positive over the illuminated MoS_2_ surface due to Fc generation and negative over the SiO_2_ substrate. Two surprising features in Figure 1C are the higher positive feedback near the edge of the MoS_2_ nanosheet and the increased negative feedback over the portion of the SiO_2_ surface adjacent to the triangle. Both features are even more prominent in the line scan of the same triangle obtained with the same tip under illumination (red curve in Figure 1D). The two current peaks due to the faster reduction of Fc^+^ can be attributed to the increased concentration of photogenerated electrons at the edge of the MoS_2_ triangle. The *i*_T_ values lower than those measured over an insulating surface (cf. red and black curves in Figure 1D) point to redox competition,^22^ i.e., the same Fc oxidation reaction occurring at both the tip and substrate surfaces (Figure 1A-ii). The redox competition response extends laterally over 2 to 3 μm on the SiO_2_ surface. These observations suggest that the separation of photogenerated electrons and holes occurs at the semiconductor–insulator interface. Photogenerated holes migrate to the SiO_2_ surface, where they oxidize Fc, while photogenerated electrons accumulate near the edge of the MoS_2_ triangle and increase the local rate of Fc^+^ reduction.

The lifetime of this electron–hole separation is relatively short. Both positive feedback and redox competition signals near the triangle edges became significantly lower in the subsequent SECM images of the same sample. This effect can be seen in the line scan recorded over the same area as the red curve in Figure 1D after a 1.5 h delay (Figure S3B, blue curve), in which the two current peaks near the edge of the triangle disappeared, and the current measured over the SiO_2_ surface became more uniform, pointing to the recombination of photogenerated electrons and holes near the SiO_2_–MoS_2_ interface.

The separation of photogenerated electrons and holes at the SiO_2_–MoS_2_ interface was observed with other thin (≤5L) MoS_2_ triangles. Figure 2A,B shows optical micrographs of bilayer and monolayer MoS_2_ triangles, respectively, with a thickness of ∼1.9 nm (Figure S2B) and ∼0.8 nm (Figure S2C). The approach curves in Figures 2C,D were recorded at points **a** (MoS_2_ surface; Figure 2A) and **b** (SiO_2_ subsurface near the edge of the triangle; Figure 2A), respectively, either in the dark (black curves) or under illumination (red curves) with the Fc mediator present in solution. Without illumination, negative feedback was observed at both locations **a** and **b**, indicating that no mediator regeneration occurred at either the MoS_2_ or SiO_2_ surface in the dark. Under illumination, positive feedback was observed over location **a** (red curve in Figure 2C) due to Fc^+^ reduction by photogenerated electrons at the MoS_2_ surface. By contrast, the tip current at point **b** became even lower under illumination (red curve in Figure 2D) due to redox competition; i.e., photogenerated holes migrated to the SiO_2_ surface and oxidized Fc to Fc^+^. Similarly, in the chopped-light transient recorded at location **c** (MoS_2_ surface in Figure 2B), the *i*_T_ increased under illumination (blue curve in Figure 2E) due to Fc regeneration, whereas at point **d** (SiO_2_ surface), the current decreased under illumination (red curve in Figure 2E), indicating that photogenerated holes migrated to SiO_2_. The amplitudes of photocurrent transients recorded over the monolayer MoS_2_ and the SiO_2_ surface decreased after a 1.5 h delay (Figure S4). This behavior is similar to that observed with a 5L MoS_2_ sample.

**Figure 2:**
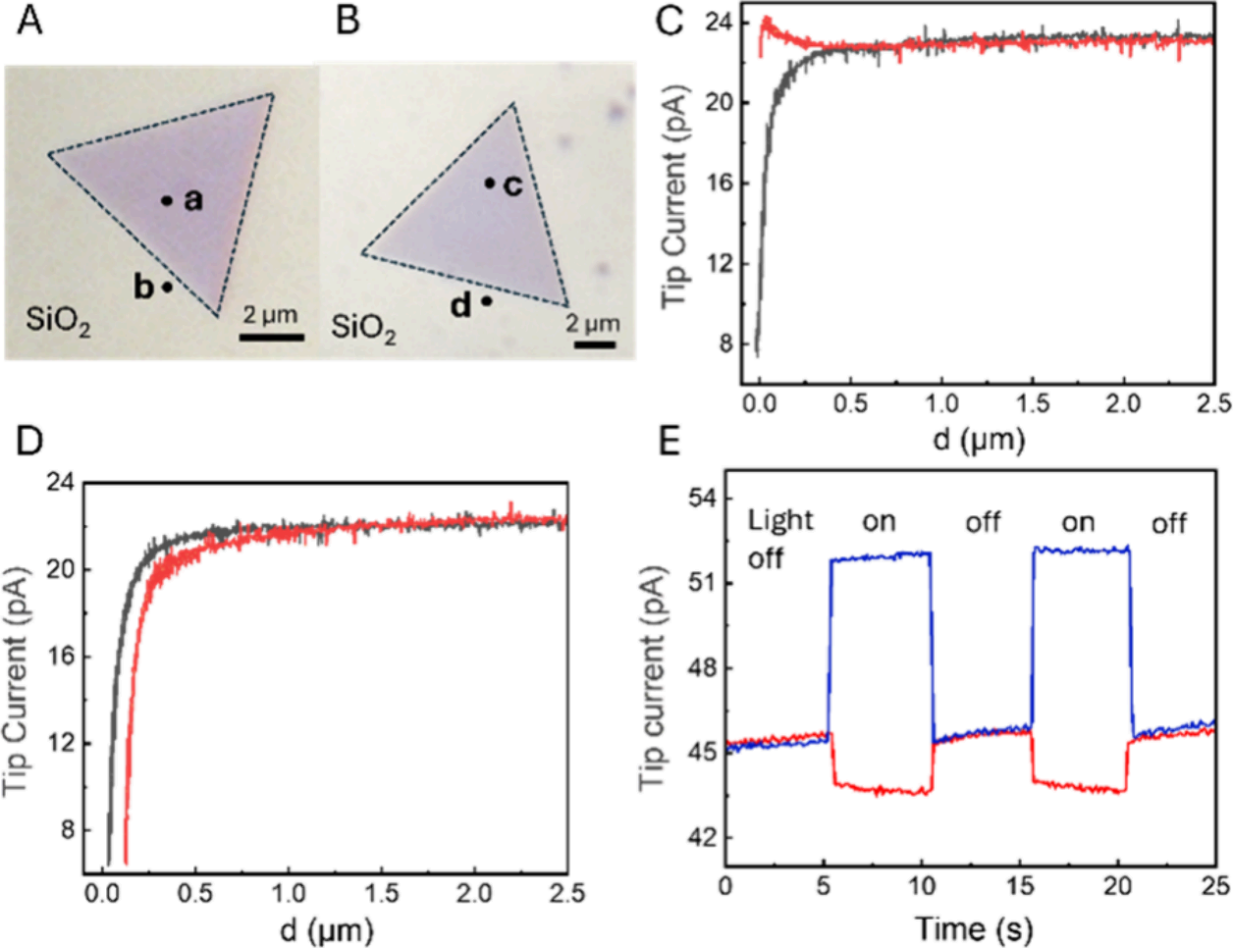
Optical microscopy and photo-SECM of monolayer and bilayer MoS_2_ triangles. Micrographs of 2L (A) and 1L (B) MoS_2_. (C, D) Approach curves measured at points **a** and **b** shown in panel A, respectively. Black curves were recorded in the dark, and red curves were recorded under illumination. (E) Chopped light current transients recorded over locations **c** (blue) and **d** (red) shown in panel B. *E*_T_ = 0.4 V vs Ag/AgCl. Solution contained 1 mM Fc in 0.1 M phosphate buffer (pH 7).

This highly reproducible charge separation behavior was observed in several sufficiently thin MoS_2_/SiO_2_ samples (Figure S7). Additional evidence of Fc oxidation at the SiO_2_ surface comes from chopped-light SECM measurements at negative and positive *E*_T_ values (Figure S8). To verify that the observed behavior is not attributable to the CVD growth conditions affecting the SiO_2_ surface, we transferred a 1L MoS_2_ triangle grown on a SiO_2_ support onto a clean SiO_2_ substrate, which was not subjected to the sulfidation procedure and performed the photo-SECM experiments that confirmed similar charge separation (Figure S9).

We used spatially resolved surface photovoltage (SPV) techniques based on Kelvin probe force microscopy (KPFM)^23,24^ to investigate the driving force for the observed charge separation at the SiO_2_–MoS_2_ interface. The topography image of the MoS_2_ triangle (Figure 3A) shows a height of about 3.3 nm, corresponding to 4L MoS_2_. The KPFM image of the same area (Figure 3B) depicts the contact potential difference (CPD) between the probe and the sample. Within the MoS_2_ region, the CPD is relatively uniform unlike the SiO_2_ surface that exhibits appreciable variations. The profile measured along the dashed line in Figure 3B revealed a gradual decrease in CPD as the scanning probe was moved laterally from the SiO_2_ to the MoS_2_ surface. The lower surface potential of SiO_2_ near the MoS_2_ region suggests that, in the dark, electrons migrate from MoS_2_ to SiO_2_, forming a space-charge region (SCR) at the interface with a width exceeding 1 μm. Control experiments performed with MoS_2_ triangles on a sapphire support did not reveal any charge separation at the MoS_2_/sapphire interface (Figure S10).

**Figure 3:**
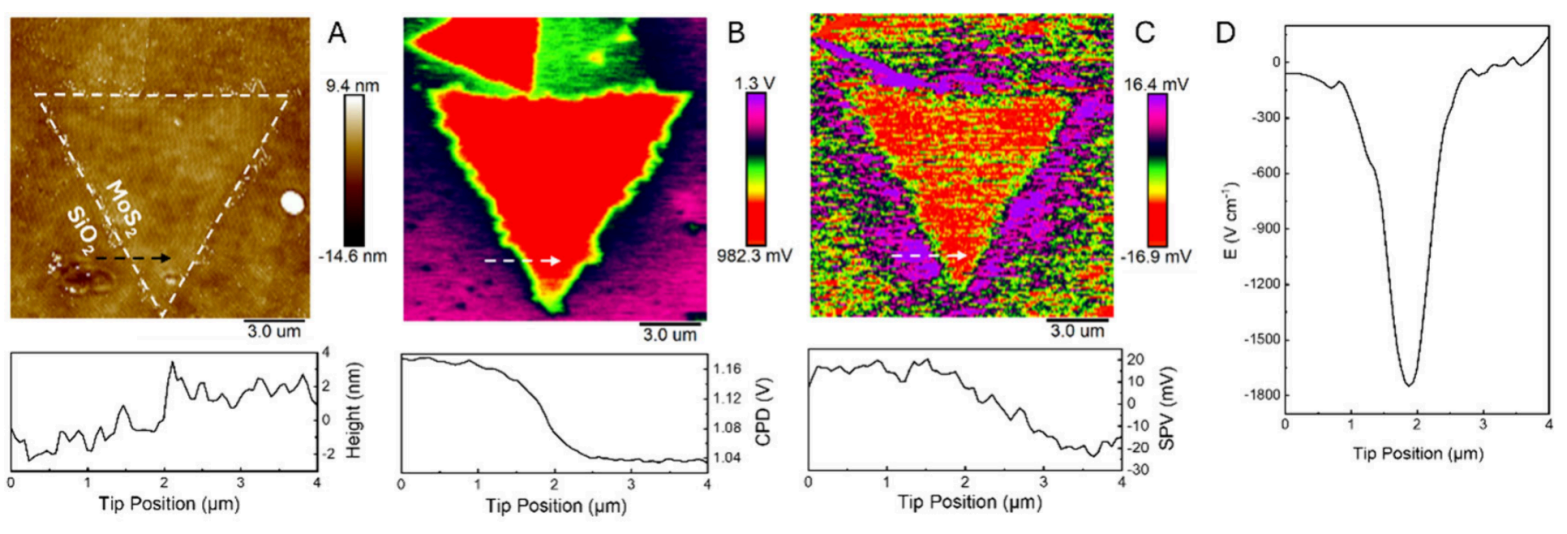
Topography (A), dark-state potential (B), and SPV (C) images of the 4L MoS_2_ triangle on the SiO_2_ support in the air. The arrows indicate the scan trajectory in the corresponding cross sections. (D) Built-in electric field at the SiO_2_–MoS_2_ interface, calculated for the same scan trajectory.

Because the energy level of the SiO_2_ conduction band (*E*_c_) is higher than that of MoS_2_, electrons should not transfer from the conduction band of MoS_2_ to that of SiO_2_.^25^ However, before equilibrium is reached, the Fermi level (*E*_F_) of MoS_2_ is higher than that of SiO_2_, providing a driving force for electron migration (Figure 4i). The defect states within SiO_2_,^11^ located near *E*_F,SiO_2__ and below *E*_c,MoS_2__ can accept electrons from MoS_2_ until equilibrium is reached. At equilibrium, MoS_2_ carries an excess positive charge, while SiO_2_ is negatively charged, creating a SCR with an electric field (*E*) directed from MoS_2_ to SiO_2_ (Figure 4ii). Based on CPD measurements performed in air, the lateral built-in electric field at the MoS_2_–SiO_2_ interface was estimated to be approximately 1.7 kV/cm (Figure 3D; see SI for discussion). This lateral built-in field, which drives the long-range separation of photogenerated carriers, is fundamentally different from the electric double-layer field normal to the surface. In a neutral aqueous solution, the negative charge of partially protonated silanol groups on SiO_2_^26^ induces additional positive charge on the adjacent MoS_2_ surface, enhancing the potential difference across the SiO_2_–MoS_2_ interface and making the built-in electric field stronger than that estimated from measurements in air.

**Figure 4:**
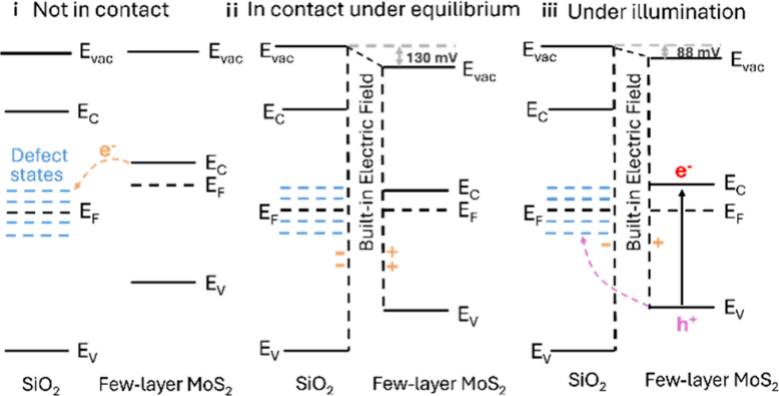
Proposed energy band diagram of the SiO_2_–MoS_2_ junction.

Under illumination, the built-in electric field becomes the primary driving force for photoexcited electron–hole separation. It drives photogenerated holes from the valence band of MoS_2_ to the defect states in SiO_2_, while electrons remain on the MoS_2_ surface (Figure 4iii). Although the electron–hole separation phenomenon gradually diminishes with time, it remains detectable after 1.5 h of illumination (see ESI for discussion).

Figure 3C shows the SPV, i.e., illuminated potential minus dark-state potential. Under illumination, a positive SPV value of approximately 18 mV was observed on SiO_2_, corresponding to the migration of photogenerated holes to the SiO_2_ surface, with a lateral migration distance exceeding 2 μm, consistent with the above SECM results (cf. Figure 1D). In contrast, MoS_2_ exhibited a negative SPV value of about −24 mV, corresponding to the accumulation of photogenerated electrons on the MoS_2_ surface.

Photogenerated charge separation is different for thicker MoS_2_ flakes. The inset in Figure 5A shows an optical micrograph of an ∼8 nm thick 10L MoS_2_ triangle (Figure S2D) whose physicochemical properties are expected to be similar to those of bulk MoS_2_. The photo-SECM image (Figure 5A) shows regions within the MoS_2_ triangle with either high positive feedback (red) or high negative feedback (dark blue). The accumulation of photogenerated electrons in the former regions enhances the reduction of Fc^+^, and the oxidation of Fc by the holes accumulated in the latter regions leads to *i*_T_ values that are lower than those measured over the SiO_2_ surface. The essentially uniform *i*_T_ over the SiO_2_ substrate suggests that photogenerated holes do not migrate from a thick MoS_2_ flake to SiO_2_. The lateral scan (Figure 5B) along the trajectory shown by the arrow in Figure 5A (inset) confirms the presence of both increased and decreased current regions within the MoS_2_ triangle under illumination and shows no significant photocurrent variations on the SiO_2_ substrate. The charge separation in this case occurred within different regions of MoS_2_.

**Figure 5:**
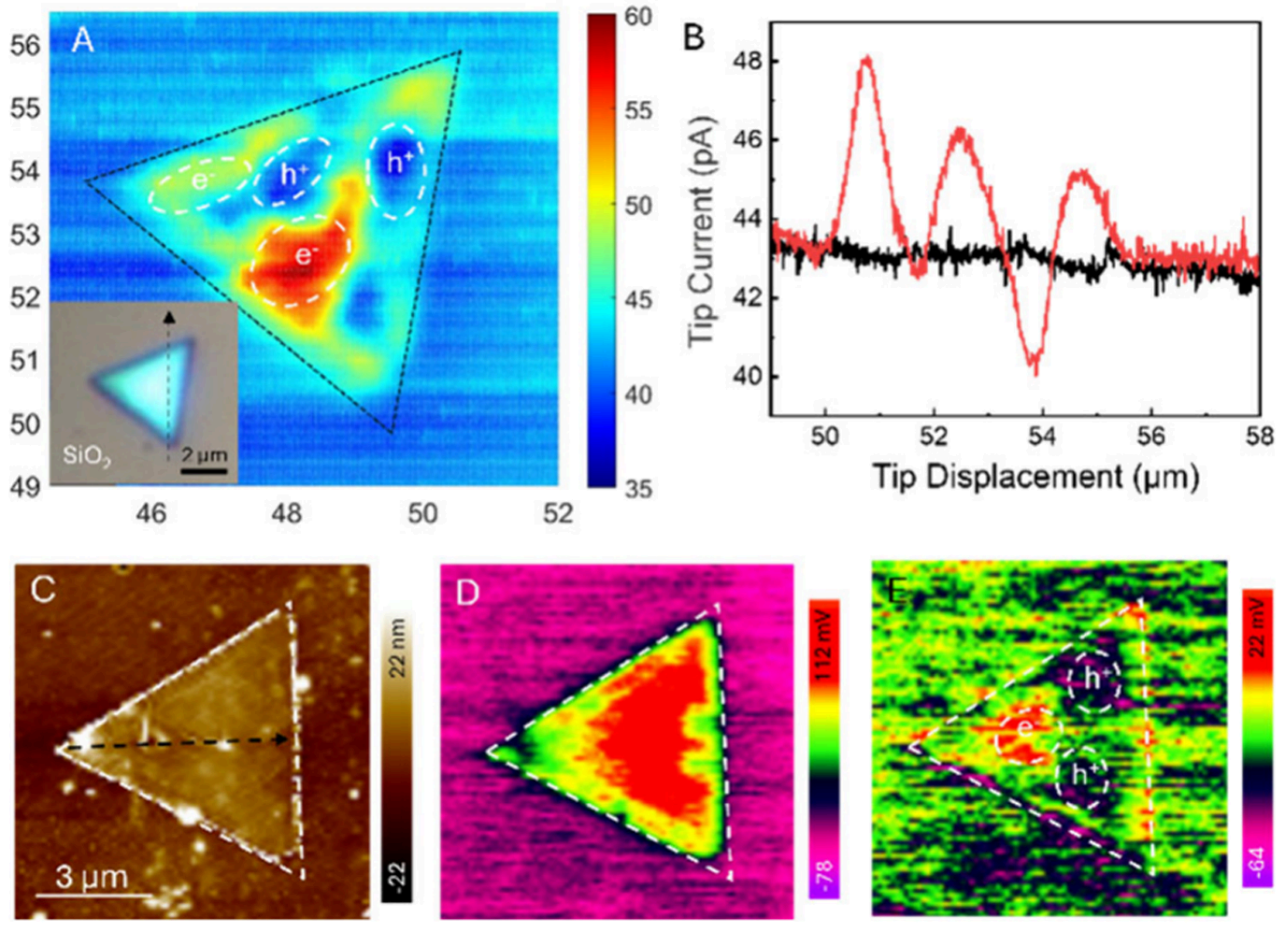
Photo-SECM (A), topography (C), dark-state potential (D), and SPV (E) images of the 10L MoS_2_ triangle on the SiO_2_ support. The arrows indicate the scan trajectory in the corresponding cross sections. The inset in panel A shows an optical micrograph of the same triangle. (B) Lateral scans along the specified trajectory (black line in panel A inset) under illumination (red curve) and in the dark (black). Solution contained 1 mM Fc in 0.1 M phosphate buffer (pH 7). *E*_T_ = 0.4 V vs Ag/AgCl. *a* = 185 nm.

The regions with e^–^ and h^+^ accumulation are still present in the photo-SECM image of the same triangle obtained after a 1.5 h delay (Figure S5A) and look similar to those in Figure 5A, though some changes in the spatial distribution of electrons and holes can be seen in the corresponding line scan (Figure S5B). In contrast to a few-layer MoS_2_ triangle, the photogenerated charge separation in a thick MoS_2_ flake is more temporally stable and does not involve significant carrier migration to the SiO_2_ surface.

Thick MoS_2_ triangles tend to exhibit defects and uneven layer growth, and their band structure varies significantly with the number of layers.^25^ The AFM topography of 10L MoS_2_ in Figure 5C shows thickness variations and pore-like depressions (Figure S6). The dark-state potential image of this triangle (Figure 5D) shows pronounced heterogeneity as compared with that of the few-layer MoS_2_ (Figure 3B). The variations in the local band structure due to different numbers of MoS_2_ layers and the presence of pores give rise to the internal electric field within a thick MoS_2_ triangle. In contrast to thin triangles, this field (rather than the built-in field at the SiO_2_–MoS_2_ interface) is a primary driving force for photogenerated charge separation in 10L of MoS_2_. Accordingly, the SPV map in Figure 5E shows the photogenerated electrons and holes accumulated within different regions of the MoS_2_ triangle and no significant charge separation at the SiO_2_–MoS_2_ interface (cf. Figure 3C), indicating that the mechanism of charge separation fundamentally changed as the number of layers increased.

In summary, high-resolution photoelectrochemical imaging of MoS_2_ triangles on a SiO_2_ support produced the first evidence of charge separation at the semiconductor/insulator interface. The photogenerated holes migrate to the SiO_2_ surface and travel laterally over distances exceeding 2 μm, while electrons remain localized within a few-layer thick MoS_2_ triangle. Thus, an insulating substrate can effectively facilitate the separation of electrons and holes in semiconductors. In thicker triangles, the internal field within MoS_2_ resulting from nonuniform morphology drives the charge separation process without significant contribution from the SiO_2_ support. This change in the charge separation mechanism requires further in-depth investigation.

## Supporting Information Available

Materials and methods, supplementary SECM and AFM images, including Figures S1–S11 (PDF)
