# Supplementary material for: Photoelectrochemical Imaging of Charge Separation between MoS2 Triangles and Insulating SiO2 Support
Source: J Am Chem Soc. 2025 Apr 23;147(18):14924–9. doi: 10.1021/jacs.5c02136 (PMC12063179; doi:10.1021/jacs.5c02136)
Supplement: Supplementary file 1 — ja5c02136_si_001.pdf [file ja5c02136_si_001.pdf]

Supporting Information for

Photoelectrochemical Imaging of Charge Separation Between MoS_2 Triangles and Insulating SiO_2 Support

Ziyuan Wang,[†,#] Qing Huang,[‡,§,#] Chenwei Ni,[‡,§] Tianyu Bo,[†,∥] Fengtao Fan,[‡,*] and Michael V. Mirkin[†,∥,*]

† *Department of Chemistry and Biochemistry, Queens College-CUNY, Flushing, NY 11367, USA.*
‡ *State Key Laboratory of Catalysis, Dalian National Laboratory for Clean Energy, iChEM, Dalian Institute of Chemical Physics, Chinese Academy of Sciences, Dalian 116023, China.*
§ *University of Chinese Academy of Sciences, Beijing 100049, China.*
∥ *The Graduate Center of CUNY, New York, NY 10016, USA.*
[*] *Corresponding Author*
Email: ftfan@dicp.ac.cn, mmirkin@qc.cuny.edu
[#] *These authors contributed equally.*

Table of contents:

Materials and Methods

Figure S1. Approach curve over a SiO_2 substrate.

Figure S2. AFM images of MoS_2 flakes with different thicknesses.

Figure S3. Optical micrograph of the 5L MoS_2 triangle and SECM lateral line scans over the same triangle.

Figure S4. Optical micrograph of the 1L MoS_2 and chopped-light SECM current transients.

Figure S5. Photo-SECM image of the 10L MoS_2 triangle on SiO_2 support and corresponding lateral scans recorded after 1.5 hours under illumination.

Figure S6. The lateral scan along the trajectory shown by the arrow in Fig. 5C.

Figure S7. Photo-SECM images of few layer MoS_2 triangles on a SiO_2 substrate.

Figure S8. Chopped-light LSV recorded at the tip held above SiO_2 and 1L MoS_2 surfaces.

Figure S9. Optical micrograph of the 1L MoS_2 triangles synthesized on a SiO_2 substrate and transferred onto a clean SiO_2 substrate and corresponding chopped-light SECM current transients.

Figure S10. Photo-SECM image of the 1L MoS_2 triangle on sapphire support.

Figure S11. Approach curves obtained with the same tip substrate before and after a 1.5-hour-long photo-SECM experiment.

Materials and Methods

Synthesis of MoS_2 Triangles. MoS_2 triangles with different thicknesses were synthesized using the chemical vapor deposition (CVD) method.[1,2] Briefly, 500 mg of sulfur powder was placed upstream in a tube furnace (Anhui BEQ), and 25 mg of molybdenum trioxide (MoO_3) was positioned in the center of the furnace. The SiO_2/Si substrates (285 nm SiO_2 thickness; P-type Si; 0.001-0.005 ohm-cm resistivity) and sapphire substrates were purchased from SixCarbon Technology, Shenzhen. The SiO_2/Si substrates were ultrasonically cleaned sequentially with acetone, isopropanol, and ethanol, followed by UV-ozone cleaning. The cleaned SiO_2/Si substrates were then placed downstream in the tube furnace. The temperatures of the sulfur powder, molybdenum trioxide, and substrates were controlled at 200°C, 780°C, and 850°C with a heating rate of 10°C/min. The reaction time was set to 5 minutes, ensuring an argon atmosphere inside the tube furnace, with an argon flow rate of 120 sccm. By controlling the reaction temperature and time, MoS_2 triangles with different thicknesses were synthesized, with the maximum reaction temperature of 900°C and the reaction time of 20 minutes.

PMMA transfer method. The PMMA anisole solution was dripped onto a 1L MoS_2 film and heated at 100 °C for 10 min. Afterwards, the PMMA-coated sample was soaked in 2 M NaOH solution for 2 hours. PMMA-MoS_2 was gradually peeled off from the SiO_2 support. After cleaning with deionized water, PMMA-MoS_2 was placed on a clean SiO_2 substrate and heated at 80 °C for 10 min. To remove PMMA, the MoS_2 sample was soaked in acetone for about 30 minutes each time by repeating this procedure three times. Finally, the sample was heated at 100°C for 10 minutes.

SECM Setup and Procedures. SECM experiments were conducted using the previously described home-built instrument[3] operating in a two-electrode configuration. The Ag/AgCl electrode served as both the reference and counter electrode, while the SECM tip acted as the working electrode. The electrolyte solution contained 1 mM ferrocenemethanol (Fc) in a 0.1 M phosphate buffer (pH = 7). In feedback mode experiments, the probe potential was set at $E_T = 0.4$ V vs. Ag/AgCl, and no external voltage was applied to the substrate. All experiments were performed at room temperature (23 ± 2 °C) inside a Faraday cage to minimize external interferences.

For photoelectrochemical experiments, the SECM instrument was integrated with an optical setup from Newport Corporation, which included an OPS-A500 500 W power supply and a 250 W HgXe lamp with a fiber bundle focusing assembly (model 77776) housed in a model 67005 lamp casing. A broad-spectrum optical fiber (model 78277, UV−vis Single Fiber Cable) with a 1 mm core diameter delivered the light. An FSQ-KG3 glass filter was used to reduce infrared radiation and prevent sample heating. A lens system coupled the optical fiber to the SECM probe, focusing light onto the back of the probe tip. The glass sheath of the nanoelectrode guided the light to the substrate beneath the probe. All SECM experiments were carried out with the light source power maintained at 250 W. A PM100D power meter (Thorlabs) equipped with an S130VC silicon photodiode power sensor was employed to measure and calibrate the light output through the optical fiber and focusing optics.[3]

SPV Measurements. Surface potential (contact potential difference, CPD) images were measured using a Kelvin Probe Force Microscope (KPFM) in air, operating in AM-KPFM mode (Bruker Dimension V SPM system). The probe was coated with Pt/Ir, with a resonance frequency of 75 Hz. The SPVM images were acquired using the lift mode, with a lift height of 20 nm and a scanning rate of 0.5 Hz, simultaneously mapping the sample's topography and surface potential. In the KPFM measurement under illumination, a 300 W xenon lamp was used as the light source. Monochromatic light was obtained via a grating, and a lens was used to focus the monochromatic light, ensuring it illuminated the sample at a specific incident angle. The surface photovoltage was defined as the difference in surface potential before and after illumination, i.e., $SPV = CPD_{light} - CPD_{dark}$.[4] The SPV signal is directly related to the separation of photogenerated charges, with its amplitude reflecting the efficiency of charge separation and its sign indicating the direction of charge transport.

Built-in electric field calculation. At the SiO_2/MoS_2 heterojunction, the contact between the two materials leads to charge transfer and band alignment, which in turn creates a built-in electric field. This field induces a spatially varying surface potential (V) that is measured by KPFM. By scanning the probe laterally over the sample along the coordinate $\boldsymbol{r}$, the built-in electric field can be directly determined using the relation:

$$\boldsymbol{E}(\boldsymbol{r}) = -\nabla V(\boldsymbol{r})$$

By differentiating the experimentally measured surface potential in Fig. 3B, we obtained the component of the built-in electric field along the r direction (shown by the arrows in Fig. 3A-C), which is approximately 1.6 kV/cm. However, the built-in electric field must be perpendicular to the heterojunction interface. The projection of the measured component back to the actual direction of the built-in electric field—perpendicular to the interface—was obtained by multiplying it by the angle correction factor $1/\cos(60°)$, yielding a corrected field strength of approximately 1.7 kV/cm, as shown in Fig. 3D.

It should be noted that different surface structures or measurement environments (such as vacuum, air, or solution) may affect the distribution of charges in the space-charge region, thereby altering the spatial profile of the surface potential and the magnitude of the built-in electric field. In our experiments, performed in air, the measured lateral potential distribution reflects only the built-in field at the SiO_2/MoS_2 interface.

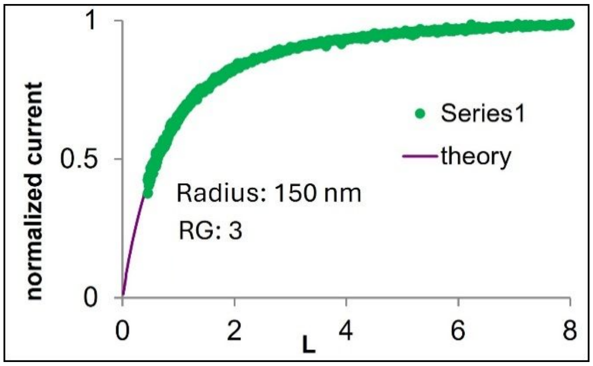

Figure S1. Experimental current-distance curve obtained with a 150-nm-radius Pt tip approaching the SiO_2 substrate in the dark (symbols) fitted to the theory (solid line). The tip current in the bulk solution, $i_{T,\infty}$ = 45.3 pA. Solution contained 1 mM Fc in 0.1 M phosphate buffer (pH 7). E_T = 0.4 V vs. Ag/AgCl. a = 165 nm.

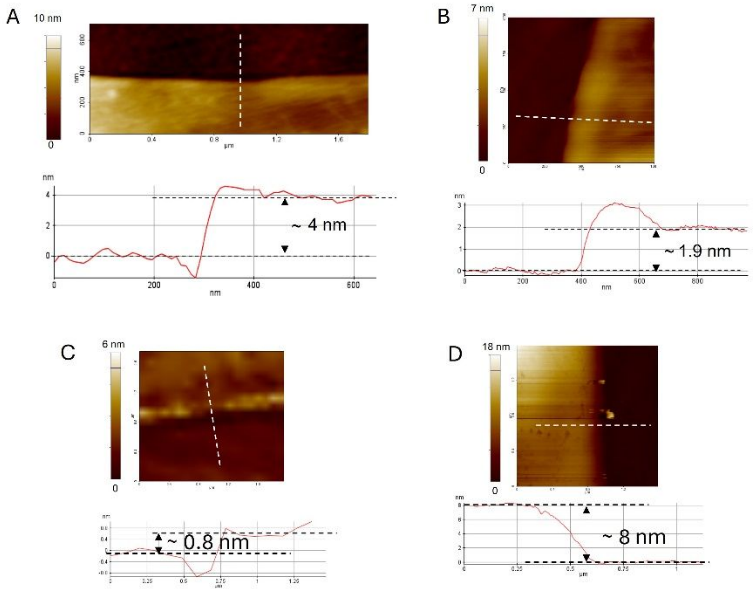

Figure S2. AFM images of MoS_2 triangles with the numbers of layers: 5L (A), 2L (B), 1L (C), and 10L (D).

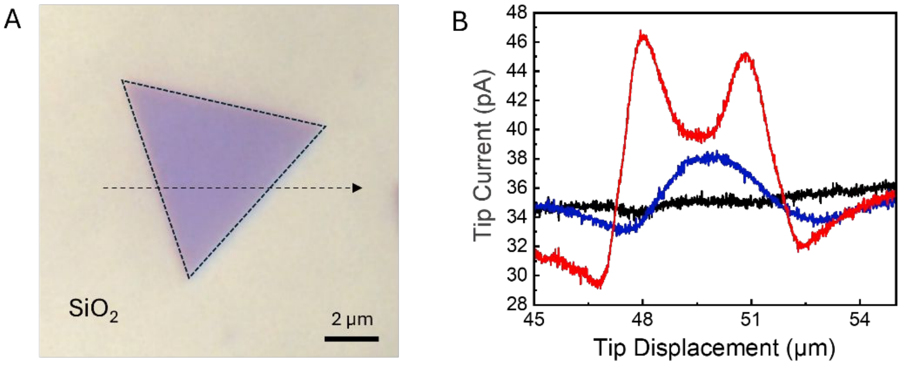

Figure S3. (A) Optical micrograph of the 5L MoS_2 flake (same as Figure 1B). (B) Lateral scans along the arrow in (A), measured in the dark (black curve), under illumination (red curve), and after 1.5 hours under illumination (blue curve). Solution contained 1 mM Fc in 0.1 M phosphate buffer (pH 7). E_T = 0.4 V vs. Ag/AgCl.

Since both water and ions in aqueous solution can act as scavengers, quickly neutralizing photogenerated carriers, the electron–hole separation can be expected to disappear quickly. The observed slow decay of charge separation under continuous illumination can be attributed to several factors specific to this system. One of them is a significant built-in electric field at the MoS_2-SiO_2 interface revealed by our SPV measurements that effectively separates electrons and holes. With holes migrating to the SiO_2 surface and electrons remaining on MoS_2, a spatial barrier is created that slows recombination, even in the presence of scavengers. Another factor is hole trapping at the SiO_2 surface by silanol-related surface states[5] stabilizing the separated charges. Once trapped, the holes may be less accessible to electrolyte ions; hence the decreased rate of charge neutralization. The interfacial water layer with a low effective dielectric constant[6] can further hinder the direct interaction between the trapped holes and the solution species, thus contributing to a persistent charge-separated state. Electrolyte cations slowly penetrate the buried interface and partially neutralize the negative surface charge, thereby reducing the effective built-in field and contributing to the observed decay in charge separation.

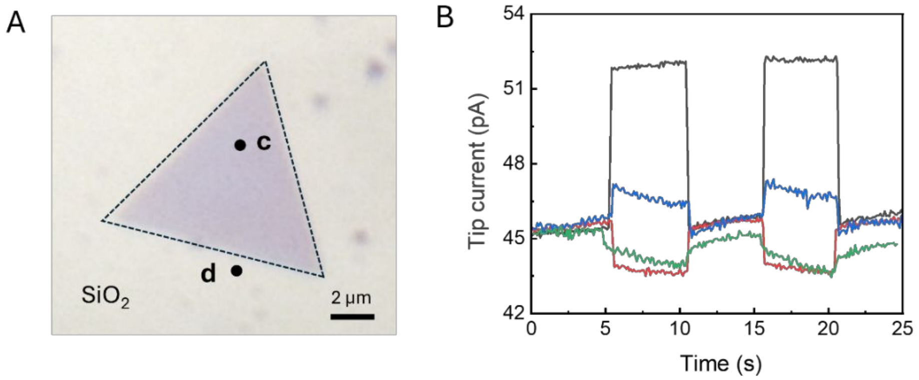

Figure S4. (A) Optical micrograph of the 1L MoS_2 flake (same as Figure 2B) and (B) chopped light-current transients recorded over point **c** on the 1L MoS_2 surface (black curve and blue curve) and point **d** on the SiO_2 substrate (red curve and green curve). The tip current was measured both initially (black curve and red curve) and after 1.5 hours of under illumination (blue curve and green curve). Solution contained 1 mM Fc in 0.1 M phosphate buffer (pH 7). E_T = 0.4 V vs. Ag/AgCl.

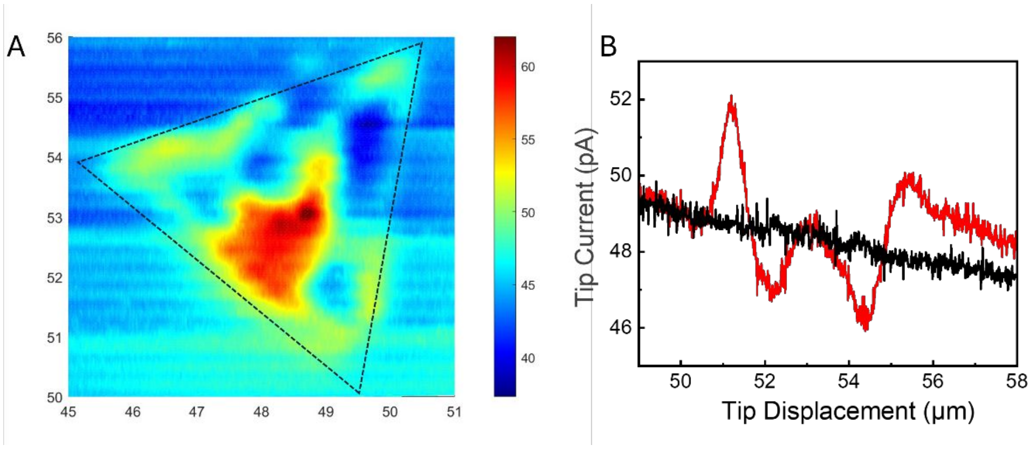

Figure S5. (A) Photo-SECM image of the 10L MoS_2 triangle on SiO_2 support (same as in Fig. 5A) and (B) corresponding lateral scans along the specified trajectory (black line in Fig. 5A inset) under illumination (red curve) and in the dark (black) taken after 1.5 hours under illumination. Solution contained 1 mM Fc in 0.1 M phosphate buffer (pH 7). E_T = 0.4 V vs. Ag/AgCl.

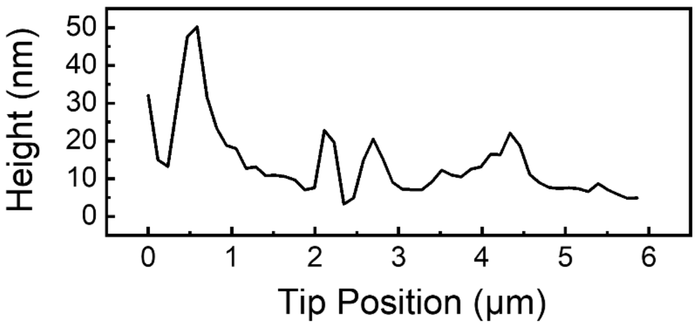

Figure S6. The lateral scan along the trajectory shown by the arrow in Fig. 5C.

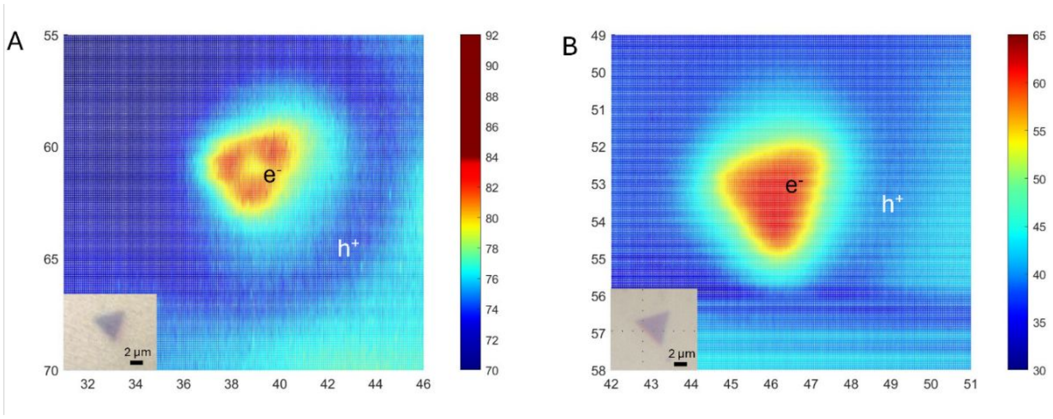

Figure S7. Photo-SECM images of a few layer MoS_2 triangles on a SiO_2 substrate. The solution contained 1 mM Fc in 0.1 M phosphate buffer (pH 7), E_T = 0.4 V vs. Ag/AgCl, and a = 165 nm.

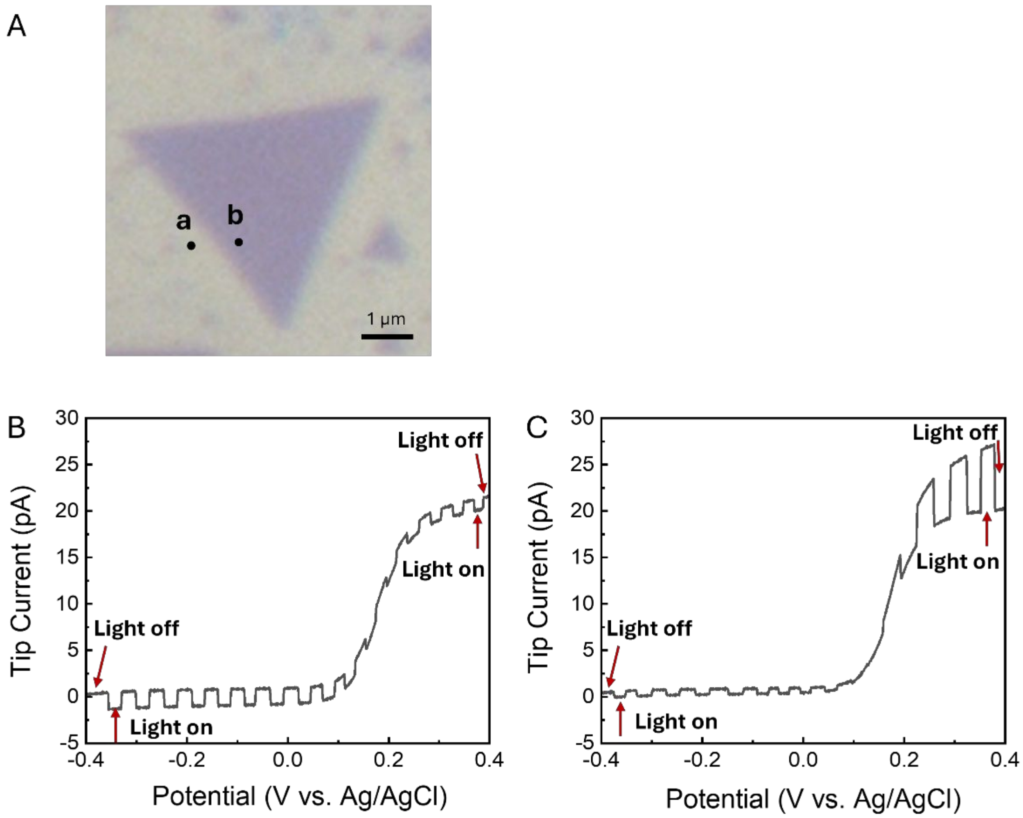

Figure S8. (A) Optical micrograph of 1L MoS_2 triangles synthesized on a SiO_2 substrate. (B, C) Chopped-light LSVs of the tip recorded over point *a* (SiO_2 surface; B) and *b* (MoS_2 surface; C) in panel A. The scan rate was 0.01 V/s. The solution contained 1 mM Fc in 0.1 M phosphate buffer (pH 7).

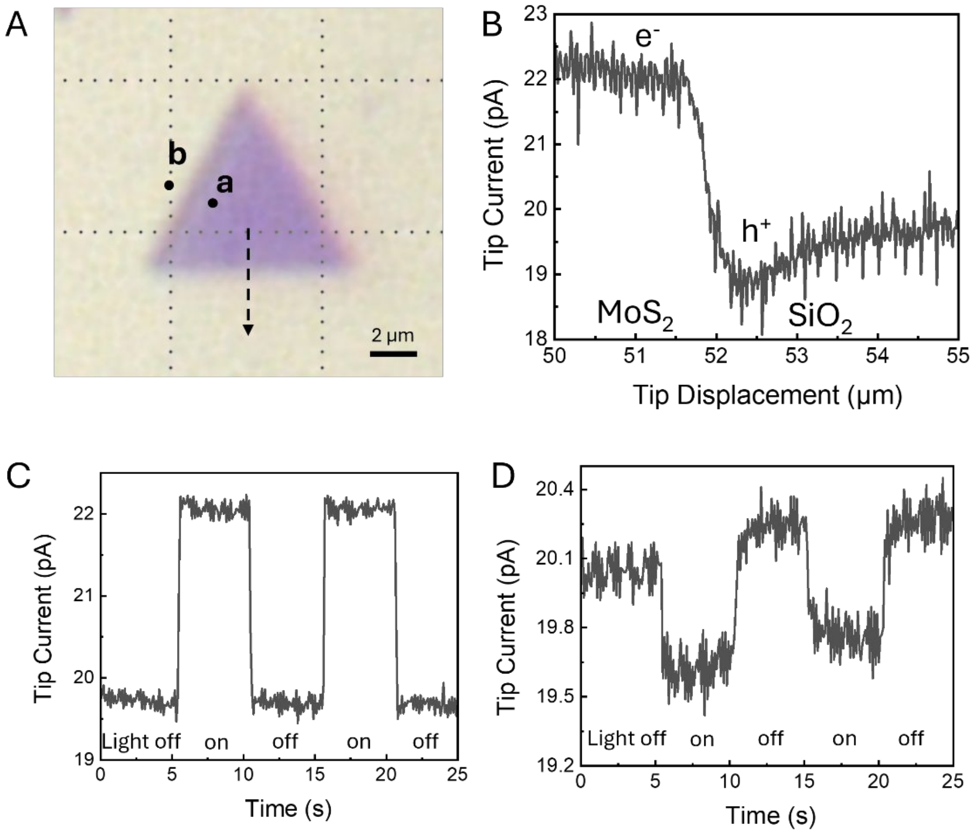

Figure S9. (A) Optical micrograph of a 1L MoS_2 triangle synthesized on a SiO_2 substrate and transferred onto a clean SiO_2 substrate using the PMMA method. (B) Lateral scan along the specified trajectory (black line in panel A) under illumination. (C) and (D) Chopped-light current transients recorded at locations **a** and **b** in panel A. E_T = 0.4 V vs. Ag/AgCl. Solution contained 1 mM Fc in 0.1 M phosphate buffer (pH 7).

In panel B, the lateral scan along the specified trajectory (black dashed line in panel A) under illumination reveals Fc^+ reduction over the MoS_2 surface (positive SECM feedback) and Fc oxidation (redox competition type signal) above the portion of SiO_2 surface adjacent to the triangle. In the chopped-light transients recorded at location *a* (above MoS_2 surface; panel A), the tip current increased under illumination due to Fc regeneration (panel C). In contrast, at point *b* (above SiO_2 surface; panel A), the current decreased under illumination, indicating that photogenerated holes migrated to SiO_2 (panel D). These findings are similar to the data shown in Figs 1 and 2, suggesting that CVD growth conditions (including sulfur incorporation) are not essential for the observed charge separation.

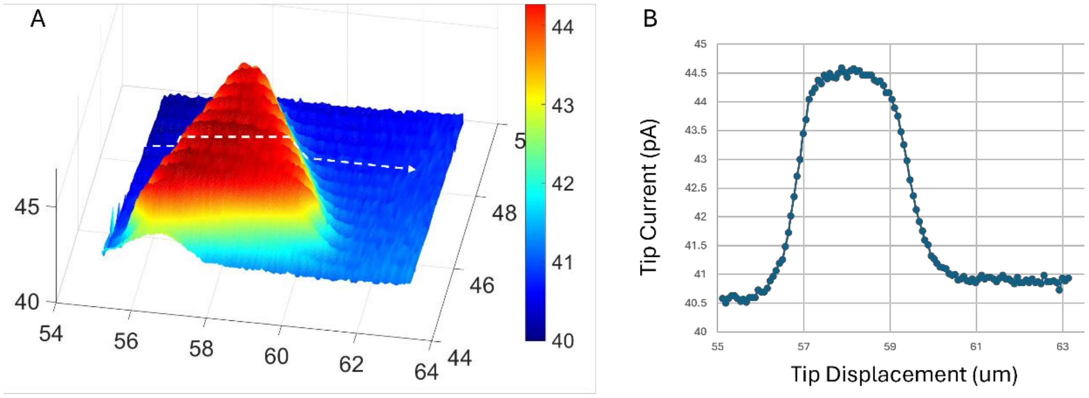

Figure S10. (A) Photo-SECM image of a 1L MoS_2 triangle on a sapphire substrate and (B) lateral scan along the white dashed line in (A). Solution contained 1 mM Fc in 0.1 M phosphate buffer (pH 7), E_T = 0.4 V vs. Ag/AgCl, and a = 165 nm.

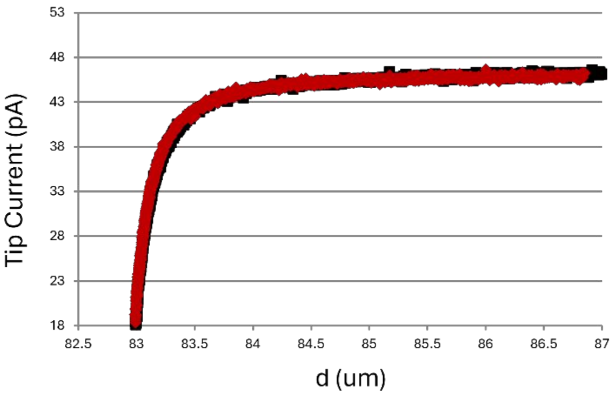

Figure S11. Approach curves obtained using the same tip and the same SiO_2 substrate in the dark before (black line) and after (red line) a 1.5-hour-long photo-SECM experiment. The same tip was used to acquire the data shown in Figs. 1 and S3.

Supplementary references

1. Lee, Y. H.; Zhang, X. Q.; Zhang, W.; Chang, M. T.; Lin, C. T.; Chang, K. D.; Yu, Y. C.; Wang, J. T.; Chang, C. S.; Li, L. J.; Lin, T. W., Synthesis of large-area MoS2 atomic layers with chemical vapor deposition. *Adv. Mater.* **2012,** *24*, 2320-5.
2. Najmaei, S.; Liu, Z.; Zhou, W.; Zou, X.; Shi, G.; Lei, S.; Yakobson, B. I.; Idrobo, J. C.; Ajayan, P. M.; Lou, J., Vapour phase growth and grain boundary structure of molybdenum disulphide atomic layers. *Nat. Mater.* **2013,** *12*, 754-9.
3. Askarova, G.; Xiao, C.; Barman, K.; Wang, X.; Zhang, L.; Osterloh, F. E.; Mirkin, M. V., Photo-scanning Electrochemical Microscopy Observation of Overall Water Splitting at a Single Aluminum-Doped Strontium Titanium Oxide Microcrystal. *J. Am. Chem. Soc.* **2023,** *145*, 6526-6534.
4. Chen, R.; Ren, Z.; Liang, Y.; Zhang, G.; Dittrich, T.; Liu, R.; Liu, Y.; Zhao, Y.; Pang, S.; An, H.; Ni, C.; Zhou, P.; Han, K.; Fan, F.; Li, C., Spatiotemporal imaging of charge transfer in photocatalyst particles. *Nature* **2022,** *610*, 296-301.
5. Pavan C.; Escolano-Casado G.; Bellomo C, Cananà S.; Tomatis M.; Leinardi R.; Mino L.; Turci F. Nearly free silanols drive the interaction of crystalline silica polymorphs with membranes: Implications for mineral toxicity. *Front. Chem.* **2023**, *10*, 1092221.
6. Fumagalli L.; Esfandiar A.; Fabregas R.; Hu S.; Ares P.; Janardanan A.; Yang Q.; Radha B.; Taniguchi T.; Watanabe K.; Gomila G.; Novoselov K.; Geim A. Anomalously low dielectric constant of confined water. *Science* **2018**, *360*, 1339-1342.
